# Electrical Properties of Membrane Phospholipids in Langmuir Monolayers

**DOI:** 10.3390/membranes11010053

**Published:** 2021-01-13

**Authors:** Anna Chachaj-Brekiesz, Jan Kobierski, Anita Wnętrzak, Patrycja Dynarowicz-Latka

**Affiliations:** 1 Faculty of Chemistry, Jagiellonian University, Gronostajowa 2, 30-387 Kraków, Poland; anna.chachaj@uj.edu.pl (A.C.-B.); anita.wnetrzak@uj.edu.pl (A.W.); 2 Department of Pharmaceutical Biophysics, Faculty of Pharmacy, Jagiellonian University Medical College, Medyczna 9, 30-688 Kraków, Poland; jan.kobierski@uj.edu.pl

**Keywords:** Langmuir monolayers, electric surface potential, dipole moments, phospholipids

## Abstract

Experimental surface pressure (π) and electric surface potential (ΔV) isotherms were measured for membrane lipids, including the following phosphatidylcholines (PCs)—1,2-dipalmitoyl-sn-glycero-3-phosphocholine (DPPC); 1,2-distearoyl-sn-glycero-3-phosphocholine (DSPC); 1,2-diarachidoyl-sn-glycero-3-phosphocholine (DAPC); and 1,2-dioleoyl-sn-glycero-3-phosphocholine (DOPC). In addition, other phospholipids, such as phosphatidylethanolamines (represented by 1,2-dipalmitoyl-sn-glycero-3-phosphoethanolamine (DPPE)) and sphingolipids (represented by N-(hexadecanoyl)-sphing-4-enine-1-phosphocholine (SM)) were also studied. The experimental apparent dipole moments ($\mu_{A}^{\exp}$) of the abovementioned lipids were determined using the Helmholtz equation. The particular contributions to the apparent dipole moments of the investigated molecules connected with their polar ($\mu_{⊥}^{p}$) and apolar parts ($\mu_{⊥}^{a}$) were theoretically calculated for geometrically optimized systems. Using a three-layer capacitor model, introducing the group’s apparent dipole moments (calculated herein) and adopting values from other papers to account for the reorientation of water molecules ($\mu_{⊥}^{w}/\varepsilon_{w}$), as well as the for the local dielectric permittivity in the vicinity of the polar ($\varepsilon_{p}$) and apolar ($\varepsilon_{a}$) groups, the apparent dipole moments of the investigated molecules were calculated ($\mu_{A}^{\mathrm{calc}}$). Since the comparison of the two values (experimental and calculated) resulted in large discrepancies, we developed a new methodology that correlates the results from density functional theory (DFT) molecular modeling with experimentally determined values using multiple linear regression. From the fitted model, the following contributions to the apparent dipole moments were determined: $\mu_{⊥}^{w}/\varepsilon_{w}=-1.8\pm 1.4\;D$; $\varepsilon_{p}=10.2\pm 7.0$ and $\varepsilon_{a}=0.95\pm 0.52$). Local dielectric permittivity in the vicinity of apolar groups ($\varepsilon_{a}$) is much lower compared to that in the vicinity of polar moieties ($\varepsilon_{p}$), which is in line with the tendency observed by other authors studying simple molecules with small polar groups. A much higher value for the contributions from the reorientation of water molecules ($\mu_{⊥}^{w}/\varepsilon_{w}$) has been interpreted as resulting from bulky and strongly hydrated polar groups of phospholipids.

## 1. Introduction

The Langmuir monolayers formed by insoluble amphiphiles at the free water surface have mainly been analyzed using the classical method based on recording surface pressure (π)–area (A) isotherms, which enables researchers to monitor changes of the physical state of film molecules upon compression [1]. Their visualization is possible with microscopic methods like Brewster angle microscopy [2] and fluorescence microscopy [3]. Changes in the electric potential (ΔV), which provide important information on the orientation of molecules at the surface, are performed less frequently. Such measurements complement the characterization of the film’s electrical properties (including dipole moments and dielectric permittivity), which play an important role in many intermolecular interactions. Their analysis provides the basis for an insight into the understanding of biomolecular processes in membranes.

A Langmuir monolayer can be considered as an array of electric dipoles of film-forming molecules situated at the air/water interface. Of particular interest are phospholipid monolayers, which provide a simplified, two-dimensional membrane model that is suitable for studying interactions [4]. Particularly important are those between the polar groups of film molecules and substances dissolved in the subphase (ions and soluble biomolecules). During interactions, the electrical surface potential of the monolayer can be decreased or increased. The measurements of such modifications are of great value as they can be used to screen drugs and check their effectiveness [5,6,7,8].

The experimental surface potential changes of a monolayer have usually been interpreted in the terms of so-called effective dipole moments ($\mu_{⊥}$). In the simplest approach, derived from the parallel plate condenser model [9], ΔV is expressed by the Helmholtz equation:

(1) $$\Delta V=\frac{\mu_{⊥}}{A\varepsilon \varepsilon_{0}}$$

where ε is the dielectric permittivity of the film, $\varepsilon_{0}$ is the dielectric permittivity of the vacuum, $\mu_{⊥}$. is the normal (to the interface) component of the dipole moment of the film molecule at the interface (note that this is different from the molecular dipole moment of the free molecule) and A is the average area occupied by the molecule at the surface (A=1/N, where N is the total number of molecules at 1 cm^2^ of the surface). The above equation applies to un-ionized molecules. For ionized ones, the double layer potential ($\psi_{0}$) must be taken into account [9,10]. The main problem in Equation (1) is the unknown value of the permittivity of the film, ε. One of the approaches assumes that ε=1, either because molecules are considered as isolated entities or because of the lack of a known value [10], however, this can be assumed only for gaseous films. Some authors have claimed that a value 5<ε<10 should be used [11]. Others suggest that for condensed monolayers, ε can be taken as 2, which is the dielectric permittivity of hydrocarbons [12]. In another approach, the unknown value of ε has been included in the so-called “apparent dipole moment” of a film molecule, $\mu_{A}=\frac{\mu_{⊥}}{\varepsilon }$ [13]. $\mu_{A}$ can be easily determined from the experimental values of ΔV as a function of A. Apart from the Helmholtz model, other approaches have been suggested in order to interpret surface potential changes (reviewed in [14,15]). A frequently used model was provided by Demchak and Fort [16], which treats the monolayer as a three-layer capacitor. In this model, the effective dipole moment of a film molecule can be divided into the contributions from the reorientation of water molecules in the monolayer, the polar and apolar part of the film molecule ($\mu_{⊥}^{w}$, $\mu_{⊥}^{p}$ and $\mu_{⊥}^{a}$, respectively), divided by their local dielectric permittivities:

(2) $$\Delta V=\frac{1}{{A\varepsilon }_{0}}\left(\frac{\mu_{⊥}^{w}}{\varepsilon_{w}}+\frac{\mu_{⊥}^{p}}{\varepsilon_{p}}+\frac{\mu_{⊥}^{a}}{\varepsilon_{a}}\right)$$

Equation (2) has been used to interpret electric surface potentials for both adsorbed [17] as well as insoluble monolayers [18]. Group dipole moments, $\mu_{⊥}^{p}$ and $\mu_{⊥}^{a}$ were calculated from bond dipole moments and angles between them, whereas local dielectric permittivities $\varepsilon_{a}$ and $\varepsilon_{p}$ were obtained by solving in pairs equations of type (2) for molecules having the same apolar parts and different polar parts, and vice versa, assuming that the contribution from the reorientation of water molecules ($\mu_{⊥}^{w}/\varepsilon_{w}$) in each pair of equations is the same. Using this procedure for adsorbed films [17] and Langmuir monolayers [18,19] formed by carboxylic acids, alcohols and their derivatives, the following values were obtained: $\varepsilon_{p}=4.2$; $\varepsilon_{a}=2.4$; ($\mu_{⊥}^{w}/\varepsilon_{w}$) = −100 ÷ −200 mD; $\varepsilon_{p}$ = 6.4; $\varepsilon_{a}$ = 2.8; ($\mu_{⊥}^{w}/\varepsilon_{w}$) = −65 mD, and $\varepsilon_{p}$ = 7.6; $\varepsilon_{a}$ = 4.2; ($\mu_{⊥}^{w}/\varepsilon_{w}$) = 25 mD, respectively. Upon analyzing surface potential changes for terphenyl derivatives [16], the following values were obtained: $\varepsilon_{p}$ = 7.6; $\varepsilon_{a}$ = 5.3; ($\mu_{⊥}^{w}/\varepsilon_{w}$) = 40 mD. Although there are some differences as regards the values of local dielectric permittivities, $\varepsilon_{p}$ is always higher than $\varepsilon_{a}$. The greatest discrepancies concern values of ($\mu_{⊥}^{w}/\varepsilon_{w}$), which are generally small, but their sign was determined to be positive or negative.

**Figure 1:**
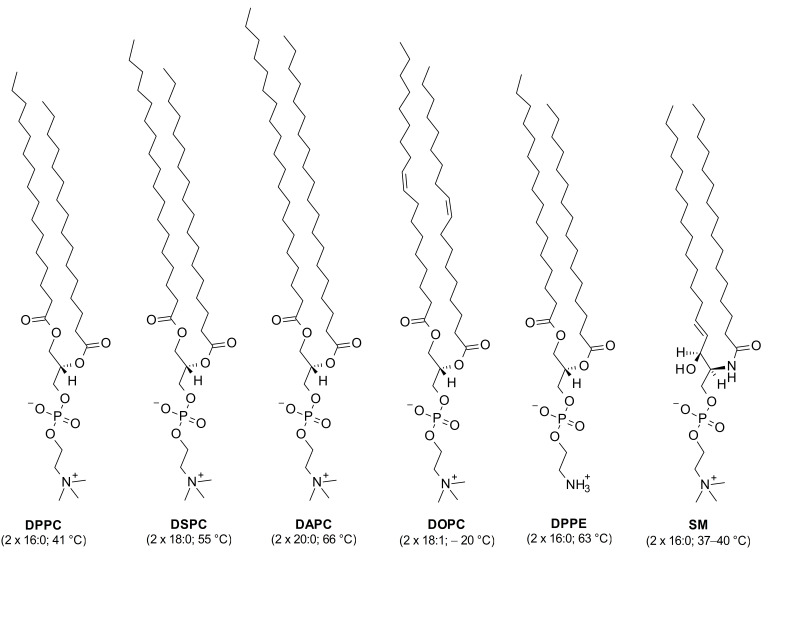
Chemical structures of the studied phospholipids together with acyl chain lengths. Chain melting temperatures of the hydrocarbon chains are given in parentheses. DPPC- 1,2-dipalmitoyl-sn-glycero-3-phosphocholine; DSPC- 1,2-distearoyl-sn-glycero-3-phosphocholine; DAPC -1,2-diarachidoyl-sn-glycero-3-phosphocholine; DOPC- 1,2-dioleoyl-sn-glycero-3-phosphocholine; DPPE- 1,2-dipalmitoyl-sn-glycero-3-phosphoethanolamine; SM- N-(hexadecanoyl)-sphing-4-enine-1-phosphocholine.

The history of measuring surface potentials of Langmuir films from membrane lipids is relatively long but there are many discrepancies in the reported values [20,21,22]. This can result from the applied approaches, laboratory procedures as well as equipment (including types of measuring electrodes used). Although nowadays this technique is routinely applied in order to analyse the electrical properties of surface films [7,8,23,24], in the literature there are results for selected, single molecules only and no systematic studies, especially for biologically important molecules, are available. The main constituent molecules of biomembranes, i.e., phospholipids, are of particular importance. Therefore, the aim of this paper was to provide the characteristics of the electrical properties of the most abundant membrane phospholipids, i.e., phosphatidylcholines (PCs), differing in acyl chain length (1,2-dipalmitoyl-sn-glycero-3-phosphocholine (DPPC); 1,2-distearoyl-sn-glycero-3-phosphocholine (DSPC); 1,2-diarachidoyl-sn-glycero-3-phosphocholin (DAPC) and unsaturation (1,2-dioleoyl-sn-glycero-3-phosphocholine (DOPC). For comparison, other phospholipids, such as phosphatidylethanolamines (represented by 1,2-dipalmitoyl-sn-glycero-3-phosphoethanolamine (DPPE) and sphingolipids (represented by N-(hexadecanoyl)-sphing-4-enine-1-phosphocholine (SM) were also investigated. The chemical structures of the studied phospholipids are shown in Figure 1. All phospholipids selected for our study have net charge zero at pH 7.

The theoretical models used so far for determining the apparent dipole moments and local dielectric permittivities were developed for very simple molecules such as carboxylic acids, alcohols and amines. The use of these models to determine the electrical properties of more complex molecules has not worked well. Therefore, an additional goal of our research was to develop a universal model to be used for molecules of any structure, based on density functional theory (DFT) modeling and multiple linear regression.

## 2. Materials and Methods

### 2.1. Surface Potential Measurements

DPPC (1,2-dipalmitoyl-sn-glycero-3-phosphocholine; 16:0 PC), DSPC (1,2-distearoyl-sn-glycero-3-phosphocholine; 18:0 PC), DAPC (1,2-diarachidoyl-sn-glycero-3-phosphocholine; 20:0 PC); DOPC (1,2-dioleoyl-sn-glycero-3-phosphocholine; 18:1 PC), egg SM (containing 86% of N-(hexadecanoyl)-sphing-4-enine-1-phosphocholine; 16:0 SM) and DPPE (1,2-dipalmitoyl-sn-glycero-3-phosphoethanolamine; 16:0 PE) in purities > 99% were supplied by Avanti Polar Lipids. All the investigated phospholipids (except for DOPC) were investigated below their chain melting temperatures (specified in Figure 1). Ethanol (98%) and spectral grade chloroform (stabilized with ethanol) were delivered by Sigma-Aldrich. Spreading solutions for Langmuir experiments were prepared by dissolving each compound in chloroform or chloroform:ethanol (9:1) with a typical concentration of 0.2–0.3 mg∙mL^−1^. In a standard experiment, 50–100 μL of the investigated solution was spread with a microsyringe (precise to ±2.5 μL). After spreading, the monolayers were left for 10 min for solvent evaporation before starting the compression at a barrier speed of 20 cm^2^/min. Deionized ultrapure water from a Millipore system with a resistivity of 18.2 MΩ∙cm, pH 7, and a surface tension of 72.8 mN/m was used as a subphase. The subphase temperature (20 °C) was controlled to within 0.1 °C using a thermostat from Julabo. Experiments were carried out with a two-barrier Langmuir 612D NIMA trough (total area 600 cm^2^) placed on an antivibration table. Surface pressure was measured with an accuracy of 0.1 mN/m using a Wilhelmy plate made of chromatography paper (Whatman Chr1) as the pressure sensor. Electric surface potential measurements were performed using a Kelvin probe (model KP2, NFT) mounted on a 612D NIMA trough. The vibrating plate was located ca. 2 mm above the water surface while the reference electrode, made from platinum foil, was placed in the water subphase. The surface potential measurements were reproducible to ±15 mV and ±2 Å^2^ per molecule. Experimental results of surface pressure–area and electric surface potential–area isotherms presented here are representative curves selected from at least three overlapping experiments.

### 2.2. Theoretical Calculations

The dipole moments and of polar $\mu_{⊥}^{p}$ and apolar $\mu_{⊥}^{a}$ parts of molecules were calculated for previously geometrically optimized systems using the Gaussian 16 software package [25]. Geometry optimization was performed by density functional theory (DFT) modeling. All calculations were performed using the B3LYP functional [26,27,28,29] with a 6-311+G(d,p) basis set [30,31] and the D3 version of Grimme’s empirical dispersion with the original D3 damping function [32]. Systems were optimized with the default convergence procedures, with no Fermi broadening.

## 3. Results and Discussion

**Figure 2:**
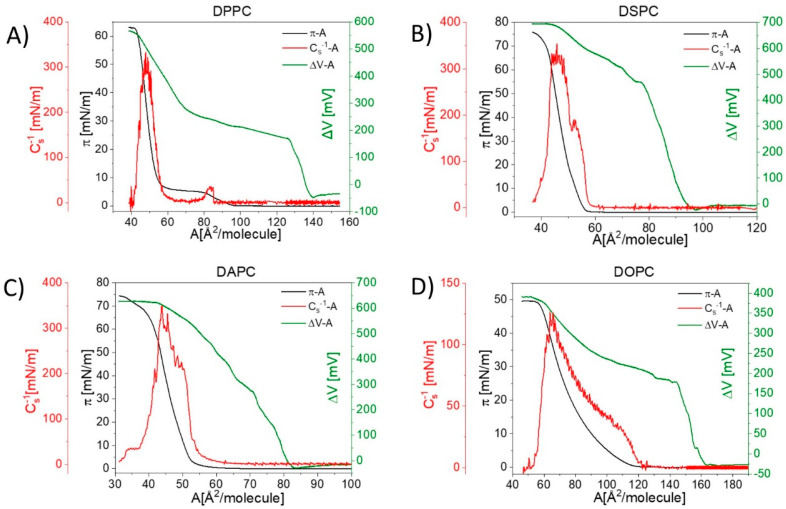
Electric surface potential change and surface pressure isotherms, together with calculated compressional moduli curves for selected phosphatidylcholines: (**A**) DPPC (1,2-dipalmitoyl-sn-glycero-3-phosphocholine), (**B**) DSPC (1,2-distearoyl-sn-glycero-3-phosphocholine), (**C**) DAPC (1,2-diarachidoyl-sn-glycero-3-phosphocholine) and (**D**) DOPC (1,2-dioleoyl-sn-glycero-3-phosphocholine).

The results of surface pressure (π) and electric surface potential changes (ΔV) versus area per molecule (A) measurements for the investigated lipids are presented in Figure 1 and Figure 2. Experimental π–A isotherms for all the studied phospholipids are in good agreement with those already published ([33] for DPPC, [34] for DSPC, [35] for DAPC, [36] for DOPC, [37] for DPPE and [38] for SM). Experimental ΔV−A curves can be characterized by two important parameters: critical area ($A_{c}$, which corresponds to the area at which ΔV is triggered off) and maximum value of surface potential (${\Delta V}_{\max}$). The change in surface potential, observed at $A_{c}$ occurs at much earlier stages of monolayer compression compared to the surface pressure lift-off area ($A_{\mathrm{lift}–\mathrm{off}}$) [10]. The onset areas of both surface pressure ($A_{\mathrm{lift}–\mathrm{off}}$) and surface potential ($A_{c}$) occur at the largest areas for the unsaturated phospholipid DOPC. This is due to the steric requirements for molecules with cis unsaturated bonds in their chains, having a coiled conformation, in contrast to saturated all-trans chains. For uncharged compounds, ΔV is approximately constant and close to zero at large areas per molecule. Upon compression, a sharp change (increase or decrease) is observed at the critical area. Changes in the slope of ΔV–A dependence reflect molecular orientation and/or conformational changes in the layer, as ΔV is proportional to the magnitude of the electrostatic field gradient normal to the subphase surface [1]. The maximum of the surface potential, ${\Delta V}_{\max}$, is defined as the value corresponding to the most vertical orientation of molecules, which usually coincides with the maximum condensation of the monolayer, characterized by the maximum compressibility modulus: $C_{s}^{-1}=-A\left(\frac{d\pi }{\mathrm{dA}}\right)$ [39]. Finally, at a collapse point, ΔV becomes approximately constant. The maximum value of the surface potential, ${\Delta V}_{\max}$, is used to calculate the apparent dipole moment $\mu_{A}^{\exp}$ of film molecules, using the following equation:

(3) $$\mu_{A}^{\exp}=\frac{\mu_{⊥}}{\varepsilon }=\varepsilon_{0}\cdot A\cdot {\Delta V}_{\max}$$

wherein $\varepsilon_{0}$ is the vacuum permittivity, ε is the monolayer permittivity (unknown) and A and $\Delta V_{\max}$ are values extracted from experimental curves corresponding to the maximum compressibility modulus. The values of $\mu_{A}^{\exp}$ for all the investigated phospholipids, together with other characteristic parameters for their monolayers, such as $A_{\mathrm{lift}–\mathrm{off}}$, $A_{c}$, $\Delta V_{\max}$ and A, are summarized in Table 1.

Let us first discuss the surface potential change curves recorded for phosphatidylcholine derivatives (Figure 2), in which the length of the apolar acyl chain or unsaturation degree is varied.

The ΔV–A curve recorded for DPPC shows a characteristic sharp increase starting at the molecular area ca. 139.0 Å^2^ from −30 to ca. 168 mV, where there is a visible inflection. Then, the surface potential rise is continued, albeit with a much smaller slope. Starting from ca. 68 Å^2^/molecule and 283 mV, the curve slope increases again. A similar sequence is observed in the π–A isotherm and can be attributed to a change in physical surface states in DPPC, i.e., a phase transition from a liquid-expanded (LE) to a liquid-condensed (LC) phase. As is well known, the LC state is more ordered than the LE state due to (i) closer molecular packing (resulting from the increased conformational order of hydrocarbon chains), and (ii) the smaller tilt angle of molecules in the surface layer [40]. The most condensed DPPC monolayer is characterized by a surface potential change equal to 509 mV, which is in agreement with previous studies (527 mV [23], 551 mV [24] and 544 mV [41]). Further elongation of acyl chains to 18 (DSPC) and 20 (DAPC) carbons influence the electrical properties of the formed monolayers. The critical area becomes shifted to smaller values: 96.2 and 82.6 Å^2^/molecule for DSPC and DAPC, respectively. This suggests that phosphatidylcholines with longer hydrocarbon chains form more tightly packed and more condensed films compared to the DPPC monolayer. The curve for DSPC is characterized by one inflection appearing at ca. 77 Å^2^/molecule and 466 mV, which suggests changes in molecular orientation in the monolayer (understood as a change in molecular angle in respect to the surface) [42]. On the other hand, the ΔV–A isotherm for DAPC shows a gradual rise without noticeable slope changes until the film collapse. The maximum surface potential values are equal to 684 mV and 614 mV for DSPC and DAPC, respectively. The DOPC molecule possesses one double bond in each of the octadecyl chains and can be compared to DSPC, which has hydrophobic chains of the same length, but both are saturated. As can be noticed, the ΔV–A curves of both phospholipids are characterized by the same shape (with one inflection). However, some differences should also be noted. Firstly, the critical area for DOPC is approximately 66 Å^2^/molecule larger as compared to DSPC. This suggests lower packing of the DOPC film, which is also confirmed by the values of the compressibility moduli. Secondly, the maximum value of the surface potential ${\Delta V}_{\max}$ is equal to 358 mV. Since the surface potential is proportional to the component of the electrostatic field vertical to the surface, it can be concluded that DOPC acyl chains in the monolayer are more disordered in comparison to DSPC. The molecular basis of this issue is due to the presence of double bonds in the cis configuration in DOPC. The configuration of unsaturated bonds affects the conformation of entire hydrocarbon chains, causing disorders (gauche defects, etc.). In contrast, the DSPC hydrocarbon backbone exists mainly in an all-trans zig-zag conformation. To sum up, the participation of hydrocarbon chains in the surface potential change of phosphatidylcholines is mainly steric, related to packing density and order, which is in agreement with previous work [43].

**Figure 3:**
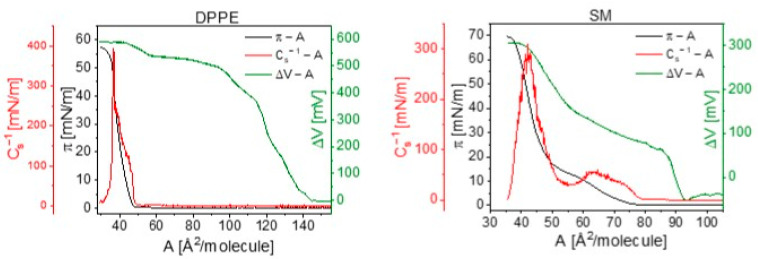
Electric surface potential change and surface pressure isotherms, together with calculated compressional moduli curves for (**A**) DPPE and (**B**) SM.

In the next step of our studies, we examined the influence of the interfacial area and polar group modification on surface potential isotherms (Figure 3).

The SM molecule contains the same polar group as the PCs (the phosphocholine group), however, its structure is different in the region of the sphingosine backbone. The π–A and ΔV–A isotherms measured for SM show an analogical course to DPPC and suggest the existence of stable liquid-expanded and liquid-condensed phases. Despite this fact, the values of the critical area (92.9 Å^2^ per molecule) and the maximum of surface potential (295 mV) for SM are significantly lower than for DPPC. Values read from the measured ΔV–A isotherm of SM are in agreement with the literature [24].

In the next part of our studies, we examined the electrical properties of monolayers composed of a representative of phosphatidylethanolamines—DPPE. The ΔV–A curve is characterized by a gradual increase, without any visible inflections, starting from molecular area 144 Å^2^ and reaching a maximum value of surface potential equal to 573 mV (in agreement with the literature data, reporting values ranging from 520 mV [18] to 589 mV [23]).

**Figure 4:**
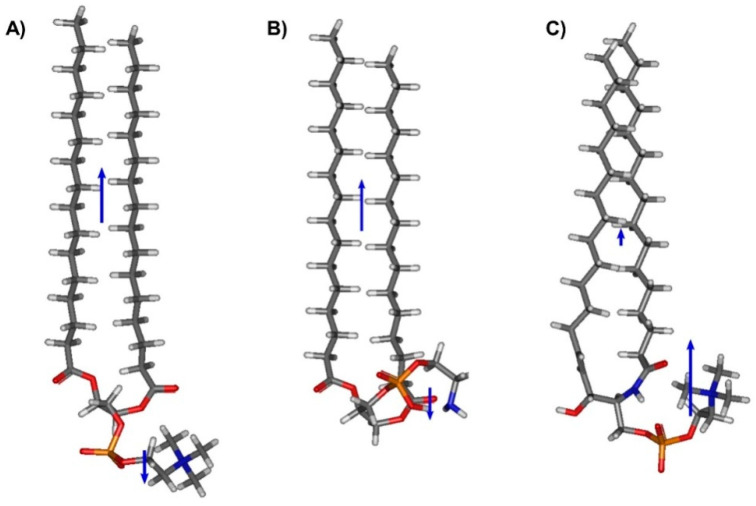
Molecular structure of selected phospholipids: DPPC (**A**), DPPE (**B**) and SM (**C**) with the directions of normal components of electrical dipole moment visualized separately for the polar group ($\mu_{⊥}^{p}$) and hydrocarbon chains ($\mu_{⊥}^{a}$). Carbon atoms are visualized in dark gray, hydrogen in bright gray, oxygen in red, nitrogen in blue and phosphorus in orange.

To enrich and interpret the results obtained from our experiments, theoretical DFT calculations of molecular conformations were performed. The values of the total dipole moments of free molecules in a vacuum ($\mu_{\mathrm{tot}}$) are compiled in Table 2. To determine the magnitude of the normal component of the electrical dipole moment, $\mu_{z}$, two different approaches were applied. Firstly, the values $\mu_{z}$ were directly read from the total dipole moment $\mu_{\mathrm{tot}}$ of the molecule in a vacuum (ε=1). Additionally, the component of the dipole moment in the plane of the interface ($\mu_{x-y}$) has also been provided. However, it should be emphasized that the calculated dipole moment corresponds to the free molecule, and not to the molecule adsorbed at the interface. Therefore $\mu_{z}$ cannot be directly compared to $\mu_{A}^{\exp}$ values obtained from experimental ΔV–A dependencies. In this approach, the molecule is treated as a single entity, without distinguishing its local parts (polar/apolar). As a result, differences in dielectric permittivity between polar and apolar parts are not taken into consideration. In the second approach, we separated the normal component of the dielectric dipole moment of a free molecule into contributions from the polar head ($\mu_{⊥}^{p}$) and hydrocarbon chains ($\mu_{⊥}^{a}$) individually (see Figure 4). The results of these theoretical calculations ($\mu_{\mathrm{tot}}$, $\mu_{x-y}$, $\mu_{z}$, $\mu_{⊥}^{p}$, $\mu_{⊥}^{a}$) are collected in Table 2.

In the next step of our calculations, we were interested in relating theoretical and experimental values, using the following equation [16], introducing the values of local dielectric permittivities ($\varepsilon_{p}$, $\varepsilon_{a}$) to the theoretically calculated normal dipole moments of the polar and apolar groups ($\mu_{⊥}^{p}$, $\mu_{⊥}^{a}$) as well as the contribution from the reorientation of water molecules ($\mu_{⊥}^{w}/\varepsilon_{w}$):

(4) $$\mu_{A}^{\mathrm{calc}}=\frac{\mu_{⊥}^{w}}{\varepsilon_{w}}+\frac{\mu_{⊥}^{p}}{\varepsilon_{p}}+\frac{\mu_{⊥}^{a}}{\varepsilon_{a}}$$

In Equation (4), there are three unknown parameters: ($\mu_{⊥}^{w}/\varepsilon_{w}$), $\varepsilon_{p}$ and $\varepsilon_{a}$. Therefore, initially, we took these values from other works [16,18,19] to see which set of values best fits the value $\mu_{A}^{\exp}$ obtained experimentally. The obtained results are presented in Table 3.

**Figure 5:**
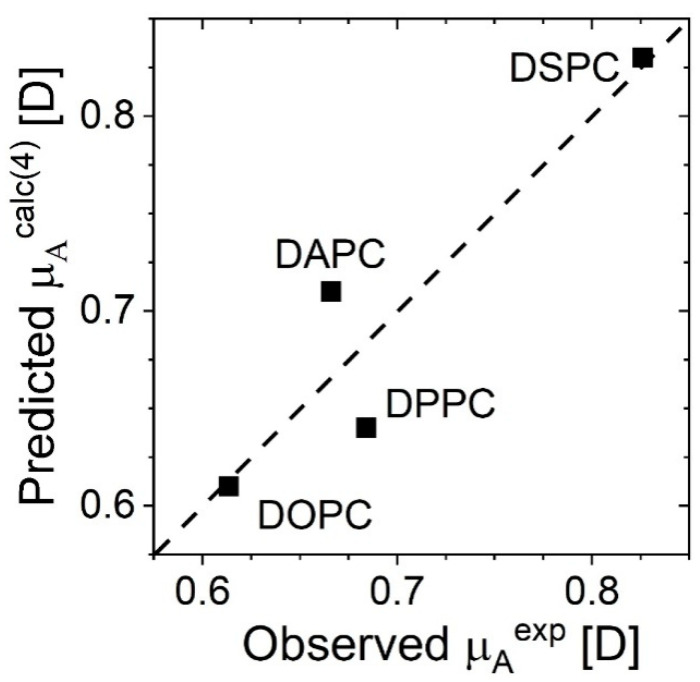
Predicted $\mu_{A}^{\mathrm{calc}\left(4\right)}\;$ versus experimental $\mu_{A}^{\exp}$ values for the studied phosphatidylcholines with the line of identity.

As can be seen, the results obtained using the literature values of parameters do not agree with the experimentally determined $\mu_{A}^{\exp}.$ Therefore, in the second step, we developed a new methodology. For a series of phosphatidylcholines DPPC, DSPC, DAPC and DOPC, multiple linear regression was used to find a model describing the relationship between the normal components of the calculated dipole moments of the polar and apolar parts of molecules and their experimental values $\left(\mu_{A}^{\exp}\right)$. The equation of the fitted model has the following form:

(5) $$\mu_{A}^{\mathrm{calc}\left(4\right)}=-1.8+0.098\cdot \mu_{⊥}^{p}+1.05\cdot \mu_{⊥}^{a}$$

which means that the determined parameters are equal to: $\mu_{⊥}^{w}/\varepsilon_{w}=-1.8\pm 1.4\;D$; $\varepsilon_{p}=10.2\pm 7.0$ and $\varepsilon_{a}=0.95\pm 0.52$. The predicted parameters show good agreement with the observed parameters, as shown in Figure 5. The R-squared statistic indicates that the fitted model explains 86% of the variability in $\mu_{A}$.

As can be seen, the second value ($\varepsilon_{a}$) is about ten times smaller compared to $\varepsilon_{p}$, which indicates that the contribution of non-polar groups to the apparent dipole moment is more significant. A similar relationship was found by other authors [16,18,19], however, this effect was not so pronounced. This can result from the fact that literature models were based on homological series of compounds with small, simple polar groups (such as −COOH, −OH, −NH_2_), whereas our approach involves bulky, zwitterionic systems. The obtained contribution from the reorientation of water molecules $\mu_{⊥}^{w}/\varepsilon_{w}$ is high, which can be explained by the ability of phosphatidylcholines to form hydrogen bonds with surrounding water dipoles. It is known that the hydration of the polar PC head groups is very high (even 11 water molecules per DPPC [44]), which may result in the formation of an organized water “quasi ice” lattice in the vicinity of a phosphocholine moiety [45]. The model developed in this paper for phosphatidylcholines cannot be applied to other phospholipids due to different hydration of polar groups (i.e., hydration for PC was determined to be 11.3 water molecules per lipid, whereas for PE it is 6.6). Although SM possesses the same polar group as PC, the presence of the hydroxyl group in the interfacial region may affect its hydration [46].

## 4. Conclusions

In this work, a systematic study on the surface potentials of Langmuir monolayers formed by the most abundant mammalian membrane phospholipids was performed to determine the values of their apparent dipole moments and to propose an improved protocol for estimating individual contributions to a three-layer capacitor model. Our methodology correlates the results from DFT molecular modeling with experimentally determined values using multiple linear regression. It can be applied to the study of other phosphatidylcholine derivatives (with similar hydration) with different hydrocarbon chain lengths and saturations. To be able to determine the parameters ($\mu_{⊥}^{w}/\varepsilon_{w}$), $\varepsilon_{p}$ and $\varepsilon_{a}$ for other kinds of phospholipids, a similar approach should be carried out for their homologous series.

## Figures and Tables

**Table 1:** Selected data read from surface pressure–area (π–A) and electric surface potential–area (ΔV–A) experimental curves measured at 20 °C, together with experimental apparent dipole moments $\mu_{A}^{\exp}$ values (uncertainty of $\pm {\Delta \mu }_{A}^{\exp}$ was obtained by exact differential method)—explanation in the text.

Phospholipid	$A_{\mathrm{lift}–\mathrm{off}}\;[{Å}^{2}/\mathrm{molecule}]$	$A_{c}\;[{Å}^{2}/\mathrm{molecule}]$	$A\;[{Å}^{2}/\mathrm{molecule}]$	$\Delta V_{\max}\;[\mathrm{mV}]$	$\mu_{A}^{\exp}[D]$	$\pm \Delta \mu_{A}^{\exp}\;[D]$
DPPC ^1^	96.2	139.0	47.8	509	0.64	0.05
DSPC ^2^	57.2	96.2	45.8	684	0.83	0.05
DAPC ^3^	58.5	82.6	43.7	614	0.71	0.05
DOPC ^4^	116.8	162.3	64.0	358	0.61	0.04
DPPE ^5^	62.2	144.2	47.7	573	0.73	0.05
SM ^6^	78.3	92.9	42.0	295	0.33	0.03

^1^ 1,2-dipalmitoyl-sn-glycero-3-phosphocholine; ^2^ 1,2-distearoyl-sn-glycero-3-phosphocholine; ^3^ 1,2-diarachidoyl-sn-glycero-3-phosphocholine; ^4^ 1,2-dioleoyl-sn-glycero-3-phosphocholine; ^5^ 1,2-dipalmitoyl-sn-glycero-3-phosphoethanolamine; ^6^ N-(hexadecanoyl)-sphing-4-enine-1-phosphocholine.

**Table 2:** Values of dipole moments and their contributions, calculated using Gaussian software for entire molecules and their selected parts in vacuum.

Phospholipid	Molecular Dipole Moment in Vacuum (D)			Group Dipole Moment (D)	
	$\mu_{tot}$	$\mu_{x-y}$	$\mu_{z}$	$\mu_{⊥}^{p}$	$\mu_{⊥}^{a}$
DPPC	13.07	13.05	0.66	−1.89	2.55
DSPC	13.01	13.00	0.55	−2.16	2.71
DAPC	13.07	13.02	1.06	−1.43	2.49
DOPC	13.04	11.88	5.39	3.40	1.99
DPPE	8.63	8.57	0.94	−1.00	1.94
SM	14.33	9.94	10.33	10.30	0.03

**Table 3:** Values of apparent dipole moments calculated from Equation (4) using the set of values of ($\mu_{⊥}^{w}/\varepsilon_{w}$), $\varepsilon_{p}$ and $\varepsilon_{a}$ equal to (1) 0.04; 7.6 and 5.3 (according to [16]); (2) −0.065; 6.4 and 2.8 (according to [18]); (3) 0.04; 7.6; 4.2 (according to [19]); (4) −1.8; 10.2; 0.95 (according to our model).

Phospholipid	Apparent Dipole Moment (D)			
	$\mu_{A}^{calc\left(1\right)}$	$\mu_{A}^{calc\left(2\right)}$	$\mu_{A}^{calc\left(3\right)}$	$\mu_{A}^{calc\left(4\right)}$
DPPC	0.32	0.60	0.44	0.68
DSPC	0.27	0.57	0.40	0.83
DAPC	0.27	0.55	0.40	0.67
DOPC	0.86	1.18	0.96	0.61
DPPE	0.27	0.47	0.37	0.13
SM	1.40	1.55	1.40	−0.78
